# Supplementary material for: A novel canis lupus familiaris reference genome improves variant resolution for use in breed-specific GWAS
Source: Life Sci Alliance. 2021 Jan 29;4(4):e202000902. doi: 10.26508/lsa.202000902 (PMC7898556; doi:10.26508/lsa.202000902)
Supplement: Supplementary file 1 [file LSA-2020-00902_TableS1.docx]

**Table S1**. Supplementary data from two storage and four nucleic acid (NA) extraction kits. Blood was preserved from four dogs (including Yella, Dog ID #7) using two different storage agents, then NA isolated using four different extraction kits. Subsets of this data were used in Tables 2 and 3. DNA 260/280 ratio, ~1.8 is considered 'pure' for DNA, ~2.0 is considered 'pure' for RNA. Expected 260/230 values are commonly in the range of 2.0–2.2.

| **Sample ID** | **Dog ID** | **Storage Agent** | **Isolation Kit** | **Isolation Volume (uL)** | **Extracted NA Conc. (ng/uL)** | **Total Extracted NA (ng)** | **Total NA**  **Normalized to Kit Isolation Vol. (ng)** | **NA**  **Quality (260/280)** | **NA**  **Quality (260/230)** | **HMW DNA**  **Yielded?** |
| --- | --- | --- | --- | --- | --- | --- | --- | --- | --- | --- |
| 12928 | 5 | EDTA | PCE | 1000 | 3.7 | 3700 | 3.7 | 2.4 | 4.6 | yes |
| 12929 | 5 | EDTA | PAXgene | 1000 | 0.36 | 360 | 0.36 | 0.55 | 0.08 | no |
| 12930 | 5 | EDTA | Nanobind | 100 | 19.5 | 1950 | 19.5 | 1.89 | 1.25 | yes |
| 12931 | 5 | EDTA | Magmax | 90 | n/a | - | - | - | - | - |
| 12932 | 5 | PAXgene reagent | PCE | 1000 | 1.11 | 1110 | 1.11 | 6.11 | -3.11 | yes |
| 12933 | 5 | PAXgene reagent | PAXgene | 1000 | 0.18 | 180 | 0.18 | 0.47 | 0.09 | no |
| 12934 | 5 | PAXgene reagent | Nanobind | 100 | 8.32 | 832 | 8.32 | 1.93 | 0.89 | yes |
| 12935 | 5 | PAXgene reagent | Magmax | 90 | 1.52 | 136.8 | 1.52 | 1.74 | 0.2 | yes |
| 12938 | 6 | EDTA | PCE | 1000 | 0.28 | 280 | 0.28 | -2.3 | -0.43 | yes |
| 12939 | 6 | EDTA | PAXgene | 1000 | 4.44 | 4440 | 4.44 | 2.21 | 9.32 | no |
| 12940 | 6 | EDTA | Nanobind | 100 | 54 | 5400 | 54 | 1.84 | 1.16 | yes |
| 12941 | 6 | EDTA | Magmax | 90 | 22.2 | 1998 | 22.2 | 1.56 | 0.23 | yes |
| 12942 | 6 | PAXgene reagent | PCE | 1000 | 5.46 | 5460 | 5.46 | 2.3 | 3.8 | yes |
| 12943 | 6 | PAXgene reagent | PAXgene | 1000 | 0.2 | 200 | 0.2 | -0.69 | -0.01 | no |
| 12944 | 6 | PAXgene reagent | Nanobind | 100 | 18.7 | 1870 | 18.7 | 1.88 | 1.85 | yes |
| 12945 | 6 | PAXgene reagent | Magmax | 90 | 11.48 | 1033.2 | 11.48 | 1.64 | 0.27 | yes |
| 12948 | 7 | EDTA | PCE | 1000 | 0.38 | 380 | 0.38 | 5.21 | -1.68 | yes |
| 12949 | 7 | EDTA | PAXgene | 1000 | 10.8 | 10800 | 10.8 | 1.98 | 6.79 | no |
| 12950 | 7 | EDTA | Nanobind | 100 | 35.3 | 3530 | 35.3 | 1.84 | 1.69 | yes |
| 12951 | 7 | EDTA | Magmax | 90 | 2.63 | 236.7 | 2.63 | 1.62 | 0.26 | yes |
| 12952 | 7 | PAXgene reagent | PCE | 1000 | 6.37 | 6370 | 6.37 | 2.2 | 3.79 | yes |

| 12953 | 7 | PAXgene reagent | PAXgene | 1000 | 6.4 | 6400 | 6.4 | 2.38 | -5.4 | no |
| --- | --- | --- | --- | --- | --- | --- | --- | --- | --- | --- |
| 12954 | 7 | PAXgene reagent | Nanobind | 100 | 11.1 | 1110 | 11.1 | 1.87 | 2.05 | yes |
| 12955 | 7 | PAXgene reagent | Magmax | 90 | 2.03 | 182.7 | 2.03 | 1.66 | 0.35 | yes |
| 12958 | 8 | EDTA | PCE | 1000 | 0.75 | 750 | 0.75 | 1.7 | 3.37 | yes |
| 12959 | 8 | EDTA | PAXgene | 1000 | 0.7 | 700 | 0.7 | 2.17 | -0.19 | no |
| 12960 | 8 | EDTA | Nanobind | 100 | 8.13 | 813 | 8.13 | 1.84 | 0.92 | yes |
| 12961 | 8 | EDTA | Magmax | 90 | 2.33 | 209.7 | 2.33 | 1.53 | 0.33 | yes |
| 12962 | 8 | PAXgene reagent | PCE | 1000 | 1.2 | 1200 | 1.2 | 2.04 | 2.59 | yes |
| 12963 | 8 | PAXgene reagent | PAXgene | 1000 | 0.59 | 590 | 0.59 | -2.48 | -0.1 | no |
| 12964 | 8 | PAXgene reagent | Nanobind | 100 | 34.4 | 3440 | 34.4 | 1.95 | 1.47 | yes |
| 12965 | 8 | PAXgene reagent | Magmax | 90 | 1.53 | 137.7 | 1.53 | 1.61 | 0.3 | yes |
