# Supplementary figures and images for: A novel canis lupus familiaris reference genome improves variant resolution for use in breed-specific GWAS
Source: Life Sci Alliance. 2021 Jan 29;4(4):e202000902. doi: 10.26508/lsa.202000902 (PMC7898556; doi:10.26508/lsa.202000902)

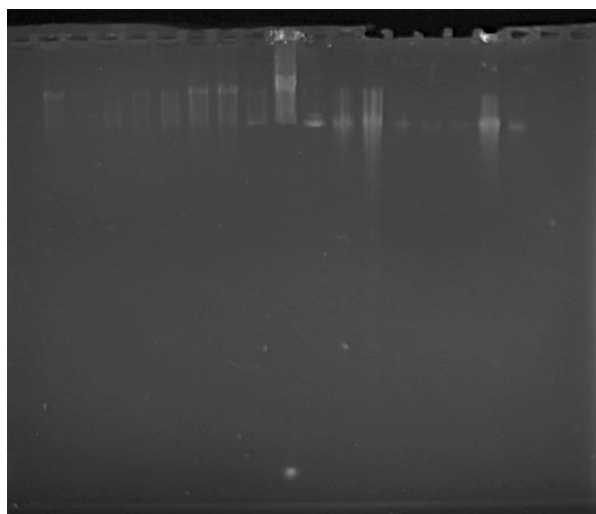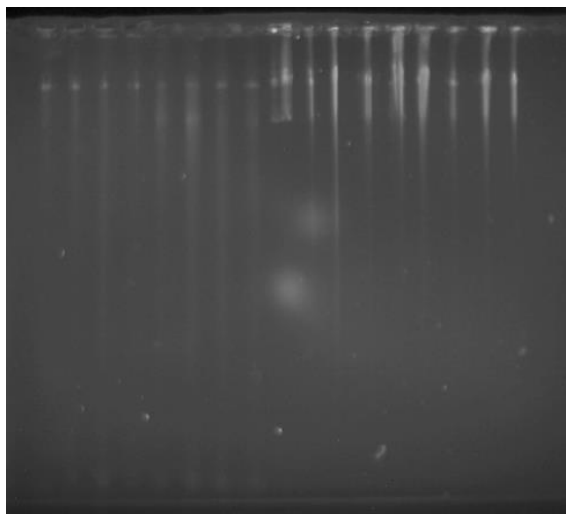

Supplement: Supplementary file 2 [file LSA-2020-00902_Supplemental_Data_1.pdf]
