# Supplementary material for: A novel canis lupus familiaris reference genome improves variant resolution for use in breed-specific GWAS
Source: Life Sci Alliance. 2021 Jan 29;4(4):e202000902. doi: 10.26508/lsa.202000902 (PMC7898556; doi:10.26508/lsa.202000902)
Supplement: Supplementary file 3 [file LSA-2020-00902_TableS2.docx]

TableS2

**Table S2**. Alignment rates and total variants of ten Labrador Retriever Illumina sequence read data sets from SRA, with additional metrics and summary statistics. CF, Boxer (CanFam3.1), GCF_000002285.3; GS, German Shepherd, GCA_008641245.1; YA, Labrador Retriever (Yella_v1.0), CP050567.1 - CP050606.1

|  | **Accession** | **Total reads in**  **SRA data set** | **Alignment rate** | | | **Total variants** | | | **Total variants with Q>=30** | | |
| --- | --- | --- | --- | --- | --- | --- | --- | --- | --- | --- | --- |
|  |  |  | **CF** | **GS** | **YA** | **CF** | **GS** | **YA** | **CF** | **GS** | **YA** |
| **Individua SRA data sets** | **SRR7107545** | 79297278 | 87.71% | 88.72% | 89.01% | 1008955 | 1022372 | 856531 | 757681 | 767110 | 610388 |
|  | **SRR7107565** | 374389398 | 94.81% | 95.40% | 95.57% | 1657855 | 1685942 | 1482554 | 1470154 | 1491641 | 1285057 |
|  | **SRR7107566** | 121998250 | 93.53% | 94.10% | 94.58% | 834633 | 852825 | 733942 | 531972 | 543237 | 436806 |
|  | **SRR7107603** | 92953674 | 94.44% | 95.02% | 95.27% | 951035 | 960583 | 872989 | 697209 | 701258 | 614153 |
|  | **SRR7107659** | 68175288 | 87.42% | 87.82% | 88.43% | 746697 | 768908 | 663296 | 399711 | 414764 | 333377 |
|  | **SRR7107891** | 194884164 | 84.58% | 84.87% | 85.22% | 966858 | 968391 | 881653 | 810122 | 810288 | 723238 |
|  | **SRR7107920** | 108772996 | 88.07% | 88.58% | 88.97% | 1196487 | 1213229 | 1022336 | 743038 | 754396 | 586261 |
|  | **SRR7107934** | 160276546 | 93.08% | 93.72% | 93.66% | 1187808 | 1210143 | 1088977 | 970907 | 989545 | 867713 |
|  | **SRR7107937** | 195746152 | 88.34% | 88.86% | 88.90% | 1247033 | 1253446 | 1130930 | 1008700 | 1012298 | 885721 |
|  | **SRR7107980** | 125140832 | 88.61% | 89.42% | 89.91% | 889701 | 913588 | 794798 | 771311 | 791011 | 670657 |
| **Statistics** | **min** | 68175288 | 84.58% | 84.87% | 85.22% | 746697 | 768908 | 663296 | 399711 | 414764 | 333377 |
|  | **max** | 374389398 | 94.81% | 95.40% | 95.57% | 1657855 | 1685942 | 1482554 | 1470154 | 1491641 | 1285057 |
|  | **median** | 123569541 | 88.47% | 89.14% | 89.46% | 987907 | 995382 | 877321 | 764496 | 779061 | 642405 |
|  | **mean** | 152163458 | 90.06% | 90.65% | 90.95% | 1068706 | 1084943 | 952801 | 816081 | 827555 | 701337 |
|  | **stdev** | 89794139 | 3.57% | 3.61% | 3.54% | 264097 | 266210 | 238776 | 292096 | 294242 | 266315 |
|  | **cov** | 0.59 | 0.04 | 0.04 | 0.04 | 0.25 | 0.25 | 0.25 | 0.36 | 0.36 | 0.38 |

Page 1
